# Interaction between Lipopolysaccharide and Gut Microbiota in Inflammatory Bowel Diseases

**DOI:** 10.3390/ijms22126242

**Published:** 2021-06-10

**Authors:** Marcello Candelli, Laura Franza, Giulia Pignataro, Veronica Ojetti, Marcello Covino, Andrea Piccioni, Antonio Gasbarrini, Francesco Franceschi

**Affiliations:** 1 Emergency Medicine Department, Fondazione Policlinico Universitario Agostino Gemelli—IRCCS, Università Cattolica del Sacro Cuore di Roma, Largo A. Gemelli 8, 00168 Rome, Italy; cliodnaghfranza@gmail.com (L.F.); giulia.pignataro@policlinicogemelli.it (G.P.); veronica.ojetti@policlinicogemelli.it (V.O.); marcello.covino@policlinicogemelli.it (M.C.); andrea.piccioni@policlinicogemelli.it (A.P.); francesco.franceschi@policlinicogemelli.it (F.F.); 2 Medical and Surgical Science Department, Fondazione Policlinico Universitario Agostino Gemelli—IRCCS, Università Cattolica del Sacro Cuore di Roma, Largo A. Gemelli 8, 00168 Rome, Italy; antonio.gasbarrini@unicatt.it

**Keywords:** inflammation, LPS, microbiota, Crohn’s disease, ulcerative colitis, IBD, Th17, Tregs, metabolic endotoxemia

## Abstract

Lipopolysaccharides (LPSs) are bacterial surface glycolipids, produced by Gram-negative bacteria. LPS is known to determine acute inflammatory reactions, particularly in the context of sepsis. However, LPS can also trigger chronic inflammation. In this case, the source of LPS is not an external infection, but rather an increase in endogenous production, which is usually sustained by gut microbiota (GM), and LPS contained in food. The first site in which LPS can exert its inflammatory action is the gut: both GM and gut-associated lymphoid tissue (GALT) are influenced by LPS and shift towards an inflammatory pattern. The changes in GM and GALT induced by LPS are quite similar to the ones seen in IBD: GM loses diversity, while GALT T regulatory (Tregs) lymphocytes are reduced in number, with an increase in Th17 and Th1 lymphocytes. Additionally, the innate immune system is triggered, through the activation of toll-like receptor (TLR)-4, while the epithelium is directly damaged, further triggering inflammation. In this review, we will discuss the importance of the crosstalk between LPS, GM, and GALT, and discuss the possible implications.

## 1. Introduction

Lipopolysaccharides (LPSs) are bacterial surface glycolipids, produced by Gram-negative bacteria. It is present in the outer membrane of most Gram-negative bacteria, which is also made up of phospholipids. LPS is composed of three domains: lipid A, the core oligosaccharide, and the O antigen. Depending on the bacteria there can be differences, however this structure is usually maintained. The main function of LPS is structural, acting as a barrier against toxic agents for the bacteria, for instance, antibiotics. It also plays an important role in stimulating the immune system, given it is one of the first bacterial components the immune system encounters [1].

Indeed, the role of LPS in stimulating inflammation has been acknowledged since the early 20th century. Pfeiffer was the first to be able to identify it and demonstrate it could cause septic shock, even though its structure was only described later. LPS can determine a very strong inflammatory reaction, mediated by its interaction with the innate compartment of the immune system [2]. Toll-like receptor (TLR)-4 is the key interlocutor of LPS, determining cytokine cascades and caspase activation [3].

LPS can determine a variety of complications other than septic shock and endotoxemia, particularly when it is chronically present at low levels. This condition is associated with metabolic endotoxemia, a condition in which alterations in the gut epithelial barrier allow microbiota-produced LPS to enter the bloodstream. Low-grade inflammation has been linked with many different diseases, such as diabetes, obesity, non-alcoholic fatty liver diseases, chronic kidney disease, and cardiovascular disease [4]. It has also been linked to immune disruption in HIV [5]. Not surprisingly, metabolic endotoxemia is typically present in inflammatory bowel diseases (IBD) [6].

While IBD does determine alterations of gut permeability, the so-called “leaky gut”, thus explaining the presence of endotoxemia in this group of persons, it is notable that LPS has been identified as a promoter of different inflammatory pathways also involved in the pathogenesis of IBD. In addition, dysbiosis associated with IBD has been associated with increased production of LPS. It appears that the interaction between LPS and IBD is, in other words, bidirectional rather than monodirectional: while IBD promotes leaky gut and endotoxemia, LPS promotes inflammation, which is pivotal in IBD. The interaction between LPS and promotion and progression of IBD is driven by immunologic pathways and microbiota modulation, which will be discussed below.

## 2. IBD: More Than One Cause

IBDs are chronic inflammatory conditions, which can typically present in two different forms: Crohn’s disease and ulcerative colitis. While the pathogenesis of IBD is not yet completely clear, it does seem that immune dysregulation is the key to the development of these disorders [7]. It appears that patients suffering from IBD present an altered Th1/Th17 lymphocyte ratio, which is common in several immune disorders [8,9], thus suggesting that local factors play an important role in the pathogenesis of Crohn’s disease and ulcerative colitis [10].

Ulcerative colitis and Crohn’s disease are both classified as inflammatory bowel diseases, yet they present some differences both in clinical presentation and pathogenic pathways. From a clinical standpoint, Crohn’s disease is characterized by the involvement of potentially any tract of the gastrointestinal tract, which tends to stabilize in time. In ulcerative colitis, on the other hand, intestinal involvement is limited to the large intestine [11]. The differences in clinical presentation are determined by differences in immune pathways: Crohn’s disease is characterized by increased levels in IL-17, IL-23, and IL-32, while in ulcerative colitis, IL-5, IL-13, IL-15, and IL-33 are upregulated. Interestingly, IL-12, IL-18, IL-21, and IL-27 are all upregulated in both diseases [12]. Differences have also been observed in terms of response to different microbial species: for instance, *Eubacterium*, *Faecalibacterium*, and *Bacteroides* stimulate a T cell response in persons with Crohn’s disease, with reduced levels of antibodies [13].

In addition, environmental factors have been studied in the pathogenesis of IBDs, particularly the importance of a healthy diet. Overall, it appears that the western diet can promote the development of such diseases, as demonstrated by the fact that their incidence is growing in those populations which are now switching to an animal-sourced diet from a plant-based one [14]. Diet works as a double risk factor through direct inflammation and microbiota mediation, which can damage the gut barrier. A damaged gut barrier allows pathogens to enter the bloodstream, in a condition known as leaky gut. If the patient does not change his dietary habits, the microbiota continues to change in a pathological sense, further enhancing systemic inflammation. Interestingly, one of the main actors in activating the immune system at a systemic level is LPS [15].

### 2.1. Immunologic Pathways

As stated above, an altered immune response is key in determining the pathogenesis of IBD. The systemic immune system is thoroughly involved, and a variety of inflammatory markers is important in diagnosing and assessing the pathology. C-reactive protein (CRP), for instance, is a highly reliable marker in determining whether the patient is responding to therapy, is in remission, or is about to relapse [16]. However, systemic inflammation is only the endpoint of complex, although localized, inflammatory pathways. It is worth noting that the gut presents one of the widest immune organs, the gut-associated lymphoid tissue (GALT). GALT represents up to 70% of the immune system [17]. It is made up of a variety of different immune cells, both belonging to the innate and the adaptive compartments. From an evolutionary standpoint, the presence of such high numbers of immune cells localized along the gut is needed to contrast all the pathogens that could potentially be ingested. However, now, the presence of so many immune cells in the gut can be potentially dangerous: for instance, alterations in the composition of GALT can promote the development of inflammatory bowel disease [18] and allergies [19].

The Peyer’s patches are one of the key components of GALT in the small bowel. They are made up of specialized T and B lymphocytes. Interestingly, most T lymphocytes present in Peyer’s patches express CD8 class I major histocompatibility complex (MHC), while only a subgroup express CD4 class II MHC [20]. The role of CD8 lymphocytes in IBD is mainly one of self-sustaining inflammation; instead, CD4 lymphocytes play a more nuanced role. Indeed, the CD4+ lymphocytes can express a wide variety of subtypes, Th1, Th2, Th9, Th17, Th22, T follicular helper (Tfh), and many kinds of T-regulatory (Treg) cells. While in normal conditions, Treg cells are prevalent in the intestine to guarantee a tolerance state, in patients with IBD, Th1 and Th17 become predominant, resulting in increased production of interferon (IFN)-γ and inflammatory interleukins (ILs), such as IL-23 and IL-12 by the Th1 subset, and IL-17 by the Th17 subset [21]. Another signature alteration of the immune system is the increased presence of tissue-resident memory T cells (TRM), which are capable of further pushing the inflammation in the gut [22]. All these changes are sustained also by antigen-presenting cells (APCs), which are stimulated in recognizing different components of the gut and the microbiota as dangerous.

B-lymphocytes also drive gut inflammation in patients suffering from IBD, producing a wide array of antibodies such as anti-pancreatic antibodies, perinuclear antineutrophil cytoplasmic autoantibodies (pANCA), anti-Saccharomyces cerevisiae mannan antibodies (ASCA), anti-erythrocyte antibodies, anti-endothelial cell antibodies, anti-bactericidal/permeability-increasing protein antibodies, and antip40 antibodies. It is worth noting that these antibodies seem capable of cross-reacting with commonly present bacteria in the intestinal lumen [20].

Overall, dysregulation of the equilibrium among the different immune components can trigger the development of IBD.

While local changes are the drivers of IBD, systemic inflammation is what allows it to progress. Indeed, it has been observed that in patients with IBD, inflammatory markers such as C-reactive protein (CRP) are increased used to monitor the progression of the disease [23]. Systemic inflammation also promotes leukocyte migration to the gut, through integrin signaling [24,25]. Cytokines also act as a chemotactic signal: TNF-α, IL-12, and IL-23 are all relevant and have been used as therapeutic targets [26,27]. IL-17 is another well-studied target in IBD, although it has been observed that targeting this pathway in other autoimmune disorders promoted recrudescence of bowel inflammation [28]. IL-6, IL-33, and IL-37 are also expressed in IBD and are also typical of autoimmune disorders, further confirming that systemic inflammation may promote the development of the disease, but that local factors are key in the way it manifests.

### 2.2. Microbiota Influence

Gut microbiota (GM) is one of the most important actors in maintaining healthy conditions, even if a definition a healthy GM has not yet been reached. However, some microbes have been associated with disease and chronic inflammation, (e.g., *E. faecalis, C. septicum*), while Short Chain Fatty Acid (SCFA)-producing bacteria exert an anti-inflammatory effect. The metabolizing properties of GM are linked to inflammation, given that their products can sometimes lead to toxic metabolites [29].

*B. fragilis* is capable, as many other microorganisms are, to interact with the immune system, stimulating the development of Tregs, while his enterotoxigenic variant may trigger the differentiation of Th17 lymphocytes [30].

GM disequilibrium has been linked to a wide array of systemic disorders, ranging from autism [31], obesity, and metabolic syndrome [32], to cardiovascular diseases [33], but it plays a critical role in intestinal disorders.

As stated above, GM can determine inflammation and, although it is not possible to define univocally a healthy GM, there are some signature alterations in IBD.

One of the most prevalent changes is the altered ratio between Firmicutes and Proteobacteria, with an increase in the latter which includes potentially pathogenic germs such as *Escherichia coli*, while the *Clostridium leptum* groups, especially *Faecalibacterium prausnitzii*, decrease [34]. Interestingly, in inflamed conditions, bacteria that determine inflammation also tend to increase. These changes are likely a consequence of the increased oxidative status, which is favorable for aero tolerant taxa such as Proteobacteria and Actinobacteria [35].

There are also some bacteria tightly associated with the presence of IBD. *Mycobacterium avium* subspecies paratuberculosis, for instance, has been linked to the development of Crohn’s disease [36], while *Fusobacterium varium* and a decrease in *Faecalibacterium prausnitzii* presence have been associated with ulcerative colitis [37,38].

Modifications in GM composition in IBD patients have been associated with recurrence: *Bacteroides, Escherichia, Eubacterium, Lactobacillus*, and *Ruminococcus* are all reduced in number shortly before relapse, as is the diversity of the GM [39].

When discussing the role of GM in the development of IBD, it is worth noting that it is not only made up of bacteria, but also of viruses, fungi, archaea, and protists, which may also play an important role. Some fungi—e.g., *Candida albicans, Aspergillus clavatus*, and, the Cystofilobasidiaceae family—have all been associated with the development of Crohn’s disease, and so has Basidiomycota/Ascomycota ratio, which has also been associated with ulcerative colitis [40,41]. *Saccharomyces cerevisiae* is also important in the pathogenesis of both Crohn’s disease and ulcerative colitis. Indeed, it is reduced in patients who develop the disease [41], despite the fact that it can, reportedly, determine dysbiosis in the same group of patients [42]. In addition, *Saccharomyces cerevisiae* antibodies seem to be present in patients with IBD, particularly Crohn’s disease, suggesting there might be an altered immunotolerance in the intestine [43]. Similarly, viruses and phages are also important in maintaining GM homeostasis. In this case, viruses belonging to the Herpes family are particularly relevant [44]. Once again, it appears that its reduction, also in terms of phages and viruses, is negatively linked to the development of IBDs in general [45].

All the described changes in the GM, fuel inflammation. GM is the main interlocutor of the immune system at a gut level and, when in homeostasis, helps promote immunotolerance, stimulating the differentiation of Tregs, while inhibiting the development of Th17 and Th1 lymphocyte subsets. When, instead, there is a dysregulation, inflammation promotes itself through a vicious circle in which GM promotes inflammation, and inflammation creates a favorable environment for pathogenic microorganisms.

The interaction between the GM and GALT has been further highlighted by the impressive results obtained through a fecal transplant in patients with both ulcerative colitis and Crohn’s disease: not only did the disease go into remission, but also the whole immune system radically changed, demonstrating that, while the GM is not causative in the development of IBD, it is a key promoter [46].

Modulating the microbiota has also proven useful in IBD. For instance, *Lactobacillus* GG has been seen to be even more effective than mesalazine in maintaining remission of ulcerative colitis [47]. Similar results have been observed when using *Bacillus subtilis*, which has been demonstrated to regulate both epithelial integrity in the colon and GM, preventing recurrences [4].

A summary of the interactions between microbiota and IBD can be found in Table 1.

## 3. LPS and IBD, a Complex Crosstalk

LPS is a widely studied inflammatory molecule. Its role in determining severe sepsis is well known and, even though antibiotics have undermined its dangers, it is still worth noting that endotoxemia is associated with a 20% increase in mortality when compared to Gram-positive sepsis [49]. LPS can also determine a chronic inflammatory status, which has been linked to a wide number of conditions. In these cases, LPS is usually produced by the GM and absorbed in the gut [4], which appears to be a negative prognostic factor in the case of endotoxemia, given that chronic endotoxemia seems to determine more severe outcomes of acute forms [50]. Chronic LPS exposure has also been linked to the development of other types of disorders, for instance Alzheimer’s disease [51].

Once again, systemic immune alterations follow more localized changes, thus LPS also directly promotes gut inflammation. It has been observed, for instance, that LPS promotes extracellular matrix protein (ECM)-1 expression in intestinal macrophages, a situation characteristic of Crohn’s disease and ulcerative colitis [52]. LPS also induces the expression of inflammatory IL, such as IL-8, and directly damages the epithelial barrier [53].

We will discuss below the different aspects of the interaction between LPS, immunity, and microbiota, then discuss how these different situations may affect the development of IBD and its progression.

### 3.1. LPS and the Immune System

LPS interacts mainly with the innate components of the immune system. The key component of this interaction is the TLR4, which triggers the expression of nuclear factor kappa-light-chain-enhancer of activated B cells (NFkB), through the Myeloid Differentiation Primary Response Gene 88 and TIR Domain-Containing Adaptor Protein [54]. NFkB is crucial to starting a variety of different inflammatory responses, mediated by tumor necrosis factor (TNF)-α and IL-12, which in turn further stimulates the NFkB pathway [55]. At the same time, the interaction between LPS and TLR4 also triggers the release of IFN-β. All these interactions stimulate the differentiation of inflammatory CD4 subtypes (particularly, Th1 and Th17). Interestingly, while APCs are the most common immune cells which present TLR4 expression, B lymphocytes can also express it if stimulated by specific molecules such as IL-4, which is produced by Th2 cells, another inflammatory subset of CD4+ lymphocytes [56]. B-lymphocytes stimulated by LPS increase their production of IL-6, TNF-α and increase their antibody production, as seen in the case of *Francisella tularensis* [57].

Overall, TLR4 induces inflammatory responses from the adaptive immune compartment through different molecules including IFN-γ, which is also involved in the activation of signal transducer and activator of transcription 1 (STAT1) [58]. The activation of this pathway further enhances inflammation. NOD-, LRR-, and pyrin domain-containing protein 3 (NLRP3) inflammasome is also activated in monocytes and induces IL-1β and IL-18 expression, critical for promoting the activation of other inflammatory molecules, such as TNF-α [59].

However, LPS stimulation of T cells can, in some subsets, instead induce an anti-inflammatory reaction: in vivo experiments in mice have shown that, in an asthmatic subset, LPS stimulation might benefit inflammation levels, improving the Th1/Th2 ratio [60]. While in patients with asthma, the reduction in inflammation can be a benefit, LPS appears to reduce inflammatory patterns in T lymphocytes also in other contexts. For instance, in viral/bacterial coinfection, LPS has been seen to reduce the number of T cells recognizing the virus, particularly natural killers (NK), suggesting that TLR-4 may actually play an important role in viral-bacterial coinfections [61].

TLR4 is also expressed at an endothelial level, where it can activate apoptosis and trigger the expression of other inflammatory cytokines [62]. In those affected by IBD, both Crohn’s disease and ulcerative colitis, TLR4 is more expressed than in healthy individuals, thus LPS activity is significantly amplified in this context [56].

**Figure 1:**
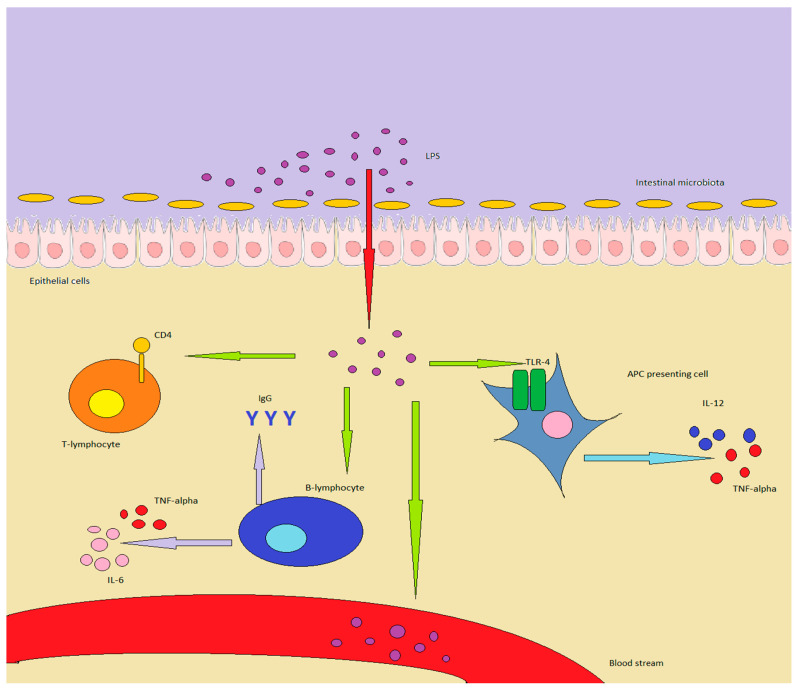
LPS in healthy individuals is produced by GM or enters through food. In inflammatory conditions or in a “leaky gut”, it can overcome the epithelial barrier, determining important effects on the immune system: in particular, APC are stimulated to produce TNF-alpha and IL-12. B-lymphocytes increase antibody production, while also producing TNF-alpha and IL-6, while T-lymphocytes are shifted towards a Th1/Th17 expression pattern. Finally, LPS can enter the bloodstream, inducing chronic or acute endotoxemia.

A summary of the interactions between immunity and LPS can be found in Figure 1.

### 3.2. LPS and Microbiota

Microbiota is the main source of LPS in healthy individuals. However, high doses of LPS are quite well-tolerated, thus it seems that LPS is relevant only once the inflammation has begun. However, this information could be partial: it has been observed that different bacteria produce different types of LPS, some of which are more likely to determine an inflammatory response. An example of this is the differences between *Bacteroides’* LPS, which is rather harmless, while LPS produced by *E. coli* is highly toxic. The consequences of this different type of exposure have been studied in Finnish, Russian, and Estonian children, and *Bacteroides*-produced LPS seems to act as an “immune-educator” in the gut [63]. LPS produced by *E. coli* also increases the levels of fecal calprotectin, which is a marker of intestinal inflammation [64]. TLR4 in cells in the intestine is not activated by LPS of typically commensal bacteria, but the same cannot be said for other organs and systems [63].

The different composition of the GM is, in other words, one of the driving factors in determining whether LPS is toxic. It is not merely LPS-producing bacteria that determine this, but also other components of the GM. One possible explanation is that certain toxic pathogens could determine a leaky gut, thus allowing even less harmful LPS to enter the bloodstream and trigger non-gut-associated cells [65].

In addition, LPS can be introduced in the gut through food: milk, for instance, can contain high quantities of it, which can determine a TLR4-driven reaction: in this case, rather than witnessing a microbiota-drive LPS activity, we observe the exact opposite, with LPS capable of determining gut inflammation, which in turn drives changes in GM [65].

Some authors suppose that orally ingested LPS is not capable of determining such damaging effects, suggesting that through this route of administration LPS is not harmful. Yet, these studies were focusing on pre- and pro-biotics, thus the results are not as widely appliable as one could hope [66].

One aspect among which most studies agree on is that, whether it be ingested orally or directly produced by one’s GM, LPS toxicity is highly dependent on dietary factors.

Western diet has been associated with the risk of developing metabolic endotoxemia, with data showing that a meal rich in fat can produce a significant increase in LPS blood levels [67]. On the other hand, a diet rich in fruit and vegetables, on the model of the Mediterranean diet, is instead associated with chronically lower LPS levels.

It is difficult to identify exactly the nature of the interaction: on the one hand, it does seem that the impact on LPS levels may be directly dependent on the diet, which could, in turn, mean that either some foods contain high levels of LPS, or that some foods induce an increased LPS production; on the other hand, an unhealthy diet may simply trigger GM alterations, which increase overall LPS production [68,69].

Both explanations are likely true to some extent, thus diet probably contributes both directly and indirectly to LPS induced endotoxemia.

The crosstalk between LPS and microbiota is also interesting in the context of IBD: indeed, the modifications described until now in individuals developing endotoxemia are the same ones identified in those who suffer from ulcerative colitis and Crohn’s disease [34]. Furthermore, LPS acts as a bridge between the GM and immunity: in particular, LPS can trigger inflammatory activation of intestinal macrophages, which shift their cytokine production from IL-10 to IL-1, IL-6, IL-8, and TNF-α [70], which impacts the immune system on a larger scale too [71,72].

### 3.3. LPS and IBD, What Do We Know

It appears clear that LPS acts as a potent immunomodulator, with interesting implications as far as the pathogenesis and progression of the disease.

As discussed, LPS acts both on the adaptive and innate immune system. In particular, it has the capacity to activate different immune pathways also involved in the pathogenesis of ulcerative colitis and Crohn’s disease. Macrophage polarization is among them: similar to T-lymphocytes, macrophages can exhibit different properties and cytokine production properties. M1 macrophages exhibit an inflammatory profile, consisting of TNF-α, IL-1α, IL-1β, IL-6, IL-12, CXCL9, and CXCL10 production, and are triggered by LPS and Th1 signaling molecules [73]. M1 polarization is also regulated by NF-kB, STAT1, STAT5, IRF3, and IRF5 pathways. The presence of M1 macrophages in the gut is typically found in both Crohn’s disease and ulcerative colitis, and LPS stimulation, as seen in murine models, is important in further promoting their expression and local inflammation [52].

M1 polarization also determines gut permeability alterations: the TLR4/NF-κB and JAK/STAT3 signaling pathways, for instance, are activated in this scenario, promoting epithelial alterations, leading to a leaky gut scenario. The blockade of this pathway does not only improve gut permeability, but also has a positive impact on persons with Crohn’s disease and ulcerative colitis, with reduced inflammation and flares [74]. In vitro models also demonstrated that interrupting the LPS signaling pathway improves the conditions of the epithelial barrier, through NF-kB inhibition [53].

While LPS promotes M1 polarization, some studies suggest that it may be important in stimulating autophagy in macrophages, which appears to play an important role in preventing IBD, particularly Crohn’s disease, for instance reducing levels of reactive oxygen species [75].

Immune system dysregulation is also important in the pathogenesis of both ulcerative colitis and Crohn’s disease, and LPS can stimulate its progression. LPS can indeed determine chronic low-grade systemic inflammation through the mechanisms of endotoxemia. It has been observed, for instance, that in persons chronically exposed to high levels of LPS, it is possible to find not only increased LPS levels but even LPS-positive bacterial extracellular vesicles. Patients with altered gut permeability at particularly high risk [6].

Also, LPS can promote the expression of fecal calprotectin, a direct consequence of intestinal inflammation. An increase in the levels of fecal calprotectin is also typical of Crohn’s disease and ulcerative colitis [76,77].

Another aspect that is worth noting is that systemic LPS induces the expression of IL-6 and other inflammatory cytokines, which can promote Crohn’s disease and ulcerative colitis [71].

Microbiota also promotes the interaction between IBD pathogenesis and progression and LPS: indeed, different bacteria present in the microbiota can produce LPS, while also directly damaging the epithelial barrier [70].

As discussed above, LPS is produced by the gut microbiota, but can also come from exogenous sources. While in normal conditions LPS does not cross the gut barrier, it has been observed that it can enter the bloodstream in conditions of altered permeability, such as Crohn’s disease and ulcerative colitis [78]. Furthermore, the presence of LPS-producing bacteria also helps alter gut permeability, resulting in a vicious circle of self-promoting inflammation [79].

## 4. Materials and Methods

Articles were identified using PubMed database, through a comprehensive search conducted by combining the following key terms: “LPS”, “gut microbiota”, “IBD”, “Crohn’s disease”, “ulcerative colitis”, “endotoxemia”, and “metabolic endotoxemia”. English-language articles were screened for relevance individually by the authors, who then compared their results, to include only the most relevant and recent articles. Case reports, letters, and opinions were excluded.

## 5. Conclusions

The role of LPS in human health is complex and probably not completely understood. While LPS allows the activation of the immune system during infections, it triggers abnormal reactions which are extremely dangerous [80], both in acute and chronic settings. In the latter, the main sources of LPS are diet and GM, thus the first site in which LPS can exert its activity is in the gut. The changes it triggers are both in GM and GALT composition: GM loses diversity, while also increasing LPS production, and GALT shifts towards an inflammatory production pattern. Interestingly, the changes in both GALT and GM are the same ones that take place in patients with ulcerative colitis and Crohn’s disease. Another interesting factor is that LPS can create direct epithelial damage in the gut, which promotes the so-called “leaky gut”, allowing endotoxemia to take place and trigger systemic immunity.

It appears that LPS is an important factor in triggering inflammation in the gut. LPS can promote the development of IBD, at least in patients who are prone to it. It also appears that reducing LPS synthesis, for instance through probiotics, allows and maintains remission, as do dietary changes. Indeed, it has been observed that the western diet promotes both ulcerative colitis and Crohn’s disease, and endotoxemia, and correcting it allows both issues to resolve.

While it is not possible to state that increased levels of LPS directly cause IBD, they can promote intestinal inflammation, a hallmark of these diseases. In particular, LPS activates pathways that are directly involved in the progression of Crohn’s disease and ulcerative colitis, particularly the NF-κB pathway, which increases levels of IL-1, IL-6, and TNF-α, among others. Some in vitro studies have focused on interrupting this inflammatory pathway, activated by LPS in IBD-like conditions, and results have been encouraging, including regression of epithelial damage after treatment with different compounds [81]. Another LPS-activated pathway that can also promote inflammation is the mTOR/STAT3 pathway, which promotes IL-6 and TNF-α production. Interrupting this pathway not only reduces the levels of inflammatory cytokines but also increases the levels of IL-10 and TGF-β1 [82].

Unfortunately, no in vivo studies have explored the issue directly, thus there needs to be further evidence to confirm this hypothesis. However, the importance of LPS does appear significant in the pathogenesis and progression of IBD.

## Figures and Tables

**Table 1:** GM and IBD.

Microbiota	Effect	Reference
*B. fragilis* enterotoxigenic variant	T-reg stimulation; Th17 stimulation.	[30]
*M. avium* subspecies paratuberculosis	Linked to the development of Crohn’s disease.	[36]
*F. varium*	Associated to IBD development.	[37]
*F. prausnitzii*	A reduced number is associated with ulcerative colitis.	[38]
*C. albicans*	Associated with the development of Crohn’s disease.	[40]
*A. clavatus*	Associated with the development of Crohn’s disease.	[40]
*S. cerevisiae*	Reduced in patients who develop IBD; antibodies present in patients with Crohn’s disease.	[40,41,42]
*Lactobacillus* GG	Maintains remission in ulcerative colitis.	[47]
*B. subtilis*	Regulates epithelial integrity, possibly preventing relapses.	[48]
*E. coli*	Increased in patients with the disease; decreases before relapse.	[34,39]
